# Obesity and Mortality, Length of Stay and Hospital Cost among Patients with Sepsis: A Nationwide Inpatient Retrospective Cohort Study

**DOI:** 10.1371/journal.pone.0154599

**Published:** 2016-04-28

**Authors:** Anh Tuan Nguyen, Chu-lin Tsai, Lu-yu Hwang, Dejian Lai, Christine Markham, Bela Patel

**Affiliations:** 1 Department of General Planning, Vinmec International Hospital, Hanoi, Vietnam; 2 Department of Emergency Medicine, Massachusetts General Hospital, Harvard Medical School, Boston, Massachusetts, United States of America; 3 Department of Epidemiology, Human Genetics and Environmental Sciences, School of Public Health, University of Texas Health Science Center at Houston, Texas, United States of America; 4 Department of Biostatistics, School of Public Health, University of Texas Health Science Center at Houston, Texas, United States of America; 5 Department of Health Promotion and Behavioral Sciences, School of Public Health, University of Texas Health Science Center at Houston, Texas, United States of America; 6 Division of Critical Care Medicine, Medical School, University of Texas Health Science Center at Houston, Texas, United States of America; University of Florida, UNITED STATES

## Abstract

**Objectives:**

The objective of this study was to examine the association between obesity and all-cause mortality, length of stay and hospital cost among patients with sepsis 20 years of age or older.

**Materials and Methods:**

It was a retrospective cohort study. The dataset was the Nationwide Inpatient Sample 2011, the largest publicly available all-payer inpatient care database in the United States. Hospitalizations of sepsis patients 20 years of age or older were included. All 25 primary and secondary diagnosis fields were screened to identify patients with sepsis using International Classification of Diseases, Ninth Revision, Clinical Modification codes. Obesity was the exposure of interest. It was one of the 29 standardized Elixhauser comorbidity measures and readily available in the dataset as a dichotomized variable. The outcome measures were all-cause in-hospital death, length of stay and hospital cost.

**Results:**

After weighting, our sample projected to a population size of 1,763,000, providing an approximation for the number of hospital discharges of all sepsis patients 20 years of age or older in the US in 2011. The overall all-cause mortality rate was 14.8%, the median hospital length of stay was 7 days and the median hospital cost was $15,917. After adjustment, the all-cause mortality was lower (adjusted OR = 0.84; 95% CI = 0.81 to 0.88); the average hospital length of stay was longer (adjusted difference = 0.65 day; 95% CI = 0.44 to 0.86) and the hospital cost per stay was higher (adjusted difference = $2,927; 95% CI = $1,606 to $4,247) for obese sepsis patients as compared to non-obese ones.

**Conclusion:**

With this large and nationally representative sample of over 1,000 hospitals in the US, we found that obesity was significantly associated with a 16% decrease in the odds of dying among hospitalized sepsis patients; however it was also associated with greater duration and cost of hospitalization.

## Introduction

Sepsis is defined as a systemic inflammatory response syndrome (SIRS) plus a documented or suspected infection; severe sepsis is sepsis plus organ dysfunction; septic shock is sepsis plus refractory hypotension or hyperlactatemia [1]. The Centers for Disease Control and Prevention (CDC) reported that hospitalizations with sepsis in the United States (US) rapidly rose from 621,000 in 2000 to 1,141,000 in 2008 [2]. In 2009, septicemia was the sixth most common principal reason for hospitalization in US with 1,665,400 inpatient stays [3]. Sepsis mortality was estimated to be 20–30% [4,5]. The number of in-hospital deaths from severe sepsis increased significantly during a 5-year period, from 154,159 deaths in 2003 to 207,427 deaths in 2007; total hospital costs for all patients with severe sepsis increased from $15.4 billion to $24.3 billion between 2003 and 2007 [6].

The World Health Organization (WHO) reported that 11% of adults aged 20 and over (i.e. 500 million) were obese worldwide in 2008 [7]. Data from the National Health and Nutrition Examination Surveys (NHANES) showed that 35.7% of adults in the US were obese in 2009–2010 [8]. According to a study on the Framingham Cohort, the duration lived with obesity was significantly associated with the risk of all-cause, cardiovascular and cancer mortality with a clear dose-response pattern [9]. Similar results were observed in other large cohort studies [10–12].

The relationship between obesity and sepsis mortality is, however, inconsistent in the literature. Some studies have suggested a protective effect of obesity on deaths from sepsis [13–16]. Two studies showed there was no significant association between obesity and sepsis mortality [17,18]. One study reported a higher risk of death in obese sepsis patients as compared to non-obese ones [19]. To our knowledge, only one published study has examined the effect of BMI on the length of hospital stay of sepsis patients and found a significant positive correlation [18]. No published studies have examined if obesity increases hospital costs for patients with sepsis. Given the public health significance of the two epidemics of obesity and sepsis, we conducted analyses to examine the association between obesity and sepsis outcomes (i.e. mortality, length of stay and hospital cost) among adults 20 years of age or older using a nationally representative inpatient database. The findings of our study will enhance the current knowledge of factors related to sepsis mortality and contribute to the healthcare management of this fatal condition.

## Materials and Methods

### Study Design

This was a retrospective cohort study.

### Study Setting

The Nationwide Inpatient Sample (NIS) is part of the Healthcare Cost and Utilization Project (HCUP), sponsored by the Agency for Healthcare Research and Quality (AHRQ), formerly the Agency for Health Care Policy and Research [20]. The NIS 2011, released in June 2013, was the latest dataset in the series of annually collected data.

The NIS is the largest all-payer inpatient care database that is publicly available in the United States, containing data from approximately 8 million hospital stays from about 1,000 hospitals sampled to approximate a 20-percent stratified sample of US community hospitals. Community hospitals are defined by the American Hospital Association to be “all non-Federal, short-term, general, and other specialty hospitals, excluding hospital units of institutions.” Included among community hospitals are specialty hospitals such as obstetrics-gynecology, ear-nose-throat, orthopedic, and pediatric institutions. Also included are public hospitals and academic medical centers. Weights are provided to calculate national estimates. Sampling of hospitals and sample weights are described elsewhere [21].

The NIS contains clinical and resource-use information that is included in a typical discharge abstract, with safeguards to protect the privacy of individual patients, physicians, and hospitals (as required by data sources). It contains more than 100 clinical and nonclinical data elements for each hospital stay such as primary and secondary diagnoses and procedures, admission and discharge status, patient demographic characteristics (e.g., sex, age, race, median household income for ZIP Code), hospital characteristics (e.g., ownership, size, teaching status), expected payment source, total charges, discharge status, length of stay and comorbidity measures.

### Human Subjects

The NIS 2011 comprised publicly available data without identifications of individual patients, physicians, and hospitals. We received the exemption to conduct this study from University of Texas Health Science Center at Houston Committee for the Protection of Human Subjects (UTHSCCPHS). The IRB number is HSC-SPH-14-0158.

### Study Subjects

We included hospitalizations of sepsis patients 20 years of age or older in the NIS 2011. All 25 primary and secondary diagnosis fields were screened to identify patients with sepsis using International Classification of Diseases, Ninth Revision; Clinical Modification (ICD-9-CM) codes. These codes were adapted from the 2011 ICD-9-CM volumes 1 & 2 for physicians professional edition by Carol J. Buck [22] and a published article on Critical Care Medicine in 2012 by Lagu et al [6]. Due to the inconsistent definitions of obesity in children and adolescents [23] and the evolving definition of sepsis in the pediatric population [24], we only studied adults 20 years of age or older. The analyzed dataset comprised 364,756 hospital discharges in the NIS 2011. In addition, subgroup analyses were conducted for patients with a primary diagnosis of sepsis, patients with severe sepsis, and patients with septic shock.

### Study measures

Obesity was the exposure of interest. Obesity was defined as BMI of 30 or higher. It was one of the 29 standardized Elixhauser comorbidity measures and readily available in the dataset as a dichotomized variable.

The outcomes of interest were all-cause in-hospital death, length of stay, hospital charge and hospital cost. Length of stay is a term which is used to calculate a patient's day of admission in the hospital till the day of discharge i.e. the number of days a patient stayed in a hospital for treatment. All-cause in-hospital death, length of stay and hospital charge were readily available variables in the dataset. Hospital charges were the total amounts billed for services, but did not reflect how much hospital services actually cost. They were converted to hospital costs by multiplying with the cost-to-charge ratios [25] based on the detailed information that hospitals send to the Center for Medicare and Medicaid Services (CMS).

A broad range of covariates potentially influencing the association between obesity and sepsis outcomes were examined. Patient variables included age, sex, locations of residence, median household income for the ZIP code, primary payer source, admission type and types of procedures patients received. Elixhauser comorbidity measures derived from ICD-9-CM codes using AHRQ’s comorbidity software [26] consist of, but are not limited to AIDS, congestive heart failure, chronic pulmonary disease, diabetes, renal failure and cancer. The validation of this comorbidity set for risk adjustment has been extensively demonstrated [27]. Hospital-level data such as number of hospital beds, ownership, location and teaching status were also included as potential covariates.

### Data Analysis

Descriptive statistics were presented as proportions for categorical variables. Age was the only continuous variable of interest and its median was reported given its skewed distribution. We tested bivariate associations between obesity, sepsis mortality, length of stay, hospital charge and hospital cost with each of the covariates using Wilcoxon rank sum tests, chi-square tests or Pearson’s correlation, as appropriate. Covariates indicating significant associations with both obesity and one of the sepsis outcomes were considered as potential confounders for the corresponding multivariate analysis. Variable selection was also based on review of medical literature [28] and clinical considerations. Multiple logistic regression was used to access the association between obesity and sepsis mortality. The association between obesity and length of stay, hospital charge and hospital cost were assessed using separate multiple linear regression models. Given the large sample size, parametric methods were robust to non-normality [29]. Therefore, we did not transform the three continuous variables length of stay, hospital charge and hospital cost to facilitate the interpretation of results even though their distributions were skewed. All the models were built using the direct approach. The direct approach is a standard modelling strategy, in which all independent variables are entered simultaneously into the model at the start of the regression analysis. This method is extensively described elsewhere [30]. Given the large sample size, we had sufficient power to adjust for all the potential confounders in the regression analyses with the rule of at least 10 events per variable [30,31]. In our analyses, there were 1007 primary sampling units (hospitals) belonging to 60 strata defined by five hospital characteristics: ownership/control, number of beds, teaching status, urban/rural location and US region. We used mixed models to account for the sampling design and the correlation of patients clustering within the same hospital. Weights were used to obtained national estimates. Variables with more than 10% of missing values were dropped from the analysis. All statistical tests were two-sided and p-values<0.05 were considered statistically significant. All statistical analyses were performed using STATA 12.0 (StataCorp, College Station, Texas).

## Results

After weighting, our sample projected to a population size of 1,763,000, providing an approximation for the number of hospital discharges of all sepsis patients 20 years of age or older in the US in 2011. The mean age of this population was 66.5 years, and women accounted for 50.6% of all hospitalizations. The proportions of severe sepsis and septic shock were 34.7% and 20.9%, respectively. Sepsis was listed as the principal diagnosis in 61% of all the sepsis cases. The overall sepsis mortality rate was 14.8% while those for severe sepsis and septic shock were 28.2% and 35.4%, respectively. The mortality rate of patients with sepsis as the principal diagnosis was 14.3%. The median hospital length of stay was 7 days (IQR = 4 to 12). The median hospital charge per sepsis hospitalization was $45,792 (IQR = $22,435 to $99,562) while the real median hospital cost was $15,917 (IQR = $8,249 to $33,947).

### Obesity status and patient characteristics

Table 1 presented the weighted estimations of the patient demographics and hospital characteristics by obesity status. Obese patients were younger, more likely to be female, more likely to reside in lower-income areas and less likely to have Medicare. There were no significant differences regarding weekend admissions and hospital number of beds between obese patients and non-obese ones. The differences in other hospital characteristics and seasons of hospitalization between the two groups were significant but minimal. Race and admission source were missing on over 10% of discharges so they were not included in the analysis.

**Table 1:** Patient demographics and hospital characteristics, according to obesity status.

	Non-obese	Obese	p-value
Patients, n	1,551,000	212,000	
**Patient characteristics**			
Age (yrs.), median (IQR)	70(56–81)	62(52–71)	<0.001
Female sex, %	49.4	59.7	<0.001
Median household income quartiles for patient's ZIP code, %			<0.001
$1-$38,999	30.4	31.1	
$39,000-$47,999	24.6	26.0	
$48,000-$62,999	24.9	25.3	
$63,000 or more	20.1	17.6	
Weekend admission, %	24.4	24.5	0.882
Insurance status, %			<0.001
Medicare	66.3	56.4	
Medicaid	10.1	12.3	
Private	17.3	24.1	
Self-pay	3.6	4.2	
Other	2.7	3.0	
Season, %			<0.001
Winter (Jan. 1–Mar. 31)	25.5	24.3	
Spring (Apr. 1–Jun. 30)	24.5	24.4	
Summer (Jul. 1–Sep. 30)	24.6	25.5	
Fall (Oct. 1–Dec. 31)	25.4	25.8	
**Hospital characteristics**			
Location/teaching status, %			0.003
Rural	11.6	9.8	
Urban non-teaching	42.7	45.3	
Urban teaching	45.7	44.9	
US region, %			<0.001
Northeast	19.0	14.1	
Midwest	21.1	24.6	
South	39.2	37.7	
West	20.7	23.6	
Control/ownership of hospital, %			0.024
Government, nonfederal	9.5	7.8	
Private, non-profit	74.7	75.9	
Private, investor-own	15.8	16.3	
Hospital number of beds, %			0.938
Small	12.6	12.7	
Medium	24.7	24.9	
Large	62.7	62.4	

Table 2 presented the weighted estimations of the comorbidities and types of procedures that patients received by obesity status. Obese patients had higher prevalence of congestive heart failure, pulmonary circulation disorders, diabetes, hypertension, chronic pulmonary diseases, liver diseases, renal failure, depression, psychoses, deficiency anemia, arthritis and hypothyroidism. However, AIDS, alcohol abuse, drug abuse, fluid and electrolyte disorders, coagulopathy, cancer, valvular diseases, paralyses and other neurological disorders were more prevalent among non-obese patients. There were no significant differences in the prevalence of chronic blood loss anemia, peptic ulcer disease and peripheral vascular disorders between obese patients and non-obese ones. Major diagnostic and therapeutic procedures were more common among the obese.

**Table 2:** Comorbidities and procedures, according to obesity status.

	Non-obese	Obese	p-value
Patients, n	1,551,000	212,000	
**Comorbidities, %**			
AIDS	0.3	0.1	<0.001
Alcohol abuse	4.4	3.2	<0.001
Deficiency anemia	33.1	35.5	<0.001
Rheumatoid arthritis/collagen vascular diseases	3.7	4.1	<0.001
Chronic blood loss anemia	1.5	1.5	0.371
Congestive heart failure	21.1	27.4	<0.001
Chronic pulmonary disease	23.7	31.4	<0.001
Coagulopathy	14.6	13.2	<0.001
Depression	10.6	14.8	<0.001
Diabetes uncomplicated	22.6	37.7	<0.001
Diabetes complicated	8.0	16.7	<0.001
Drug abuse	3.3	2.9	0.006
Hypertension	54.8	67.6	<0.001
Hypothyroidism	12.6	15.3	<0.001
Liver disease	5.4	6.3	<0.001
Lymphoma	2.1	1.2	<0.001
Fluid and electrolyte disorders	55.6	54.2	0.002
Metastatic cancer	5.2	2.4	<0.001
Paralysis	7.2	5.3	<0.001
Other neurological disorder	15.4	10.1	<0.001
Peripheral vascular disorders	9.6	9.7	0.682
Psychoses	5.2	7.2	<0.001
Pulmonary circulation disorders	5.1	8.1	<0.001
Renal failure	25.2	30.6	<0.001
Solid tumor without metastasis	4.1	2.5	<0.001
Peptic ulcer disease excluding bleeding	0.05	0.04	0.268
Valvular diseases	6.7	5.9	<0.001
Weight loss	20.4	16.3	<0.001
**Procedure**			<0.001
No major procedure	81.8	77.6	
Major diagnostic (Dx) procedure	0.6	0.7	
Major therapeutic (Tx) procedure	16.4	20.3	
Both major Dx and Tx procedures	1.2	1.4	

### The association between obesity and sepsis mortality

After adjustment, obesity was significantly associated with a 16% decrease in the odds of dying among hospitalized sepsis patients (adjusted OR = 0.84; 95% CI = 0.81 to 0.88). This association remained significant as we examined subcategories of sepsis: sepsis as the principal diagnosis, severe sepsis or septic shock (Table 3).

**Table 3:** The association between obesity and sepsis mortality.

Definition of sepsis	Odds ratio^a^	95% Confidence interval	p-value
All cases of sepsis	0.84	0.81 to 0.88	<0.001
Sepsis as the principal diagnosis	0.81	0.76 to 0.85	<0.001
Severe sepsis	0.79	0.75 to 0.82	<0.001
Septic shock	0.82	0.78 to 0.86	<0.001

^*a*^Adjusted for age, sex, median household income, weekend admission, seasons, insurance status, hospital control/ownership, hospital number of beds, hospital location/teaching status, type of procedures, 28 comorbid conditions.

### The association between obesity and length of stay

After adjusting for potential confounders, the average hospital length of stay was slightly longer for obese sepsis patients as compared to non-obese ones (adjusted difference = 0.65 day; 95% CI = 0.44 to 0.86). This association remained significant in the same direction as we examined subcategories of sepsis such as sepsis as the principal diagnosis, severe sepsis or septic shock and stratified by vital status (alive/dead) at discharge (Table 4).

**Table 4:** The association between obesity and hospital length of stay.

Definition of sepsis & vital status at discharge	Beta-coefficient^b^ (Difference in LOS)	95% Confidence interval	p-value
All cases of sepsis			
Alive and dead	0.65	0.44 to 0.86	<0.001
Alive only	0.70	0.49 to 0.91	<0.001
Sepsis as the principal diagnosis			
Alive and dead	0.54	0.37 to 0.71	<0.001
Alive only	0.55	0.37 to 0.72	<0.001
Severe sepsis			
Alive and dead	0.61	0.38 to 0.84	<0.001
Alive only	0.68	0.42 to 0.94	<0.001
Septic shock			
Alive and dead	0.74	0.40 to 1.08	<0.001
Alive only	0.66	0.28 to 1.05	<0.001

^*b*^Adjusted for age, sex, median household income, weekend admission, seasons, insurance status, hospital control/ownership, hospital number of beds, hospital location/teaching status, type of procedures, 28 comorbid conditions.

### The association between obesity and hospital charge and hospital cost

Hospital charge information represented the amount that hospitals billed for services, but did not reflect how much hospital services actually cost. Therefore, we built separate models for each as the outcome variable. After adjusting for potential confounders, the average hospital charge was significantly higher for obese sepsis patients as compared to non-obese ones (adjusted difference in hospital charge per hospitalization = $8,723; 95% CI = $5,573 to $11,873). The adjusted difference in hospital cost was $2,927 (95% CI = $1,606 to $4,247). This association remained significant in the same direction as we examined subcategories of sepsis: sepsis as the principal diagnosis, severe sepsis or septic shock. The largest differences in hospital charge and hospital cost between the two groups were observed in patients with septic shock (Table 5).

**Table 5:** The association between obesity and hospital charges and hospital costs.

Definition of sepsis & hospital charge or cost	Beta-coefficient^c^ (Difference in hospital charge or cost)	95% Confidence interval	p-value
All cases of sepsis			
Hospital charge	8,723	5,573 to 11,873	<0.001
Hospital cost	2,927	1,606 to 4,247	<0.001
Sepsis as the principal diagnosis			
Hospital charge	8,035	5,417 to 10,652	<0.001
Hospital cost	2,792	1,707 to 3,878	<0.001
Severe sepsis			
Hospital charge	8,161	4,812 to 11,510	<0.001
Hospital cost	2,168	128 to 4,208	0.037
Septic shock			
Hospital charge	11,428	6,553 to 16,304	<0.001
Hospital cost	3,551	614 to 6,488	0.018

^*c*^Adjusted for age, sex, median household income, weekend admission, seasons, insurance status, hospital control/ownership, hospital number of beds, hospital location/teaching status, type of procedures, 28 comorbid conditions.

## Discussion

In this nationally representative sample of hospitalized sepsis patients in over 1,000 hospitals, we found that obese patients had significantly lower mortality as compared to non-obese ones. This result was robust in our sensitivity analyses with subgroups of sepsis patients (i.e. sepsis as the principal diagnosis, severe sepsis, septic shock). The significantly association between obesity (or higher BMI) and mortality among sepsis patients was observed in several other studies [15,32]. The adjusted odds ratio of 0.84 (95% CI = 0.81 to 0.88) indicated a significant association of small magnitude between obesity and mortality. Some smaller studies have found a similar relationship but could not prove it to be statistically significant [13,14,16,17].

The biological mechanism for this obesity paradox in sepsis mortality could be explained by the suppression of the inflammatory response in obese patients. This is a topic of controversy in current literature. Some studies showed that inflammation and obesity were positively correlated [33–35]. However, other studies on both humans and mouse models have indicated that the serum concentrations of inflammatory cytokines such as IL-6, TNF-α, and MCP-1 were lower in obese subjects as compared to non-obese ones [15,36–38]. Sepsis is a systemic inflammatory response; hence it is the host, not the germ that drives the pathogenesis of sepsis [28]. With a compromised immune system, obese sepsis patients are likely to have less severe inflammatory response, less tissue damage, less septic shock and subsequently better survival. In addition, energy storage may play a role in decreased mortality among obese patients in need of critical care [39].

The obesity paradox has been reported in patients with stroke, myocardial infarction, heart failure, renal disease, diabetes or intensive care patients [40–44]. Some authors argued that the apparent obesity paradox was due to selection bias [45,46]. They explained that selection bias was a result of restricting the study population to a certain disease affected by the exposure and sharing common causes with the outcome. However, this type of bias was small in our study since the magnitude of the association between obesity (the exposure) and sepsis (the disease conditioned on) was not large [16,17,47]. In addition, the association between obesity and mortality observed in our study was restricted to patients with sepsis only. It should not be misinterpreted to be applicable to the general population.

The difference in average length of stay for sepsis hospitalizations between obese and non-obese patients was less than a day (0.65 day; 95% CI = 0.44 to 0.86). It might lead to a higher hospital cost. On average an obese sepsis case cost $2,927 more than a similar non-obese one. It was unclear whether the increase in hospital cost and length of stay had any plausible relationship with the increased survival in obese patients as compared to non-obese ones.

Several limitations should be noted in our study. First, the NIS is administrative data without detailed information about specific symptoms, signs and laboratory test results. Therefore, we could not control for these variables in our analysis. However, we have reasonable surrogate indicators about the patients’ condition such as their diagnoses, validated comorbidities and procedures. Moreover, our sensitivity analyses comprising different levels of sepsis severity showed that the results did not significantly differ by severity level. Therefore, residual confounding may be minimal. Second, it was possible that obesity was under-reported in administrative data as it is not required for billing purposes. Patients who were obese might have been misclassified as being non-obese. Though it could not be confirmed, such misclassification was assumed to be non-differential between people who were alive or dead at discharge. Subsequently, it would likely bias the measures of association towards the null. Therefore, such a bias would make our significant findings more conservative. Third, the information about patients’ BMI was not available in the NIS dataset so we could not further analyze the results according to different grade of obesity. Fourth, the NIS did not contain patient identifiers so we could not assess hospital readmissions or track long-term outcomes after discharge.

## Conclusion

With this large and nationally representative sample of over 1,000 hospitals in the US, we found that obesity was significantly associated with a 16% decrease in the odds of dying among hospitalized sepsis patients; however it was also associated with greater duration and cost of hospitalization. These results contribute to the emerging body of evidence regarding the obesity paradox. Future studies should examine the mechanism through which obesity reduces sepsis mortality so that new interventions might be implemented accordingly to increase survival of this fatal disease.
